# Construction of a novel MK-4 biosynthetic pathway in *Pichia pastoris* through heterologous expression of *Hs*UBIAD1

**DOI:** 10.1186/s12934-019-1215-9

**Published:** 2019-10-10

**Authors:** Xiaowen Sun, Hui Liu, Peng Wang, Li wang, Wenfeng Ni, Qiang Yang, Han Wang, Hengfang Tang, Genhai Zhao, Zhiming Zheng

**Affiliations:** 1 Key Laboratory of High Magnetic Field and Ion Beam Physical Biology, Hefei Institutes of Physical Science, Chinese Academy of Sciences, Hefei, 230031 Anhui People’s Republic of China; 2 University of Science and Technology of China, Hefei, 230026 Anhui People’s Republic of China

**Keywords:** *Pichia pastoris*, Aromatic prenyltransferase, *Hs*UBIAD1, Geranylgeranyl diphosphate synthase, Menaquinone

## Abstract

**Background:**

With a variety of physiological and pharmacological functions, menaquinone is an essential prenylated product that can be endogenously converted from phylloquinone (VK_1_) or menadione (VK_3_) via the expression of *Homo sapiens* UBIAD1 (*Hs*UBIAD1). The methylotrophic yeast, *Pichia pastoris*, is an attractive expression system that has been successfully applied to the efficient expression of heterologous proteins. However, the menaquinone biosynthetic pathway has not been discovered in *P. pastoris*.

**Results:**

Firstly, we constructed a novel synthetic pathway in *P. pastoris* for the production of menaquinone-4 (MK-4) via heterologous expression of *Hs*UBIAD1. Then, the glyceraldehyde-3-phosphate dehydrogenase constitutive promoter (P_*GAP*_) appeared to be mostsuitable for the expression of *Hs*UBIAD1 for various reasons. By optimizing the expression conditions of *Hs*UBIAD1, its yield increased by 4.37 times after incubation at pH 7.0 and 24 °C for 36 h, when compared with that under the initial conditions. We found *Hs*UBIAD1 expressed in recombinant GGU-23 has the ability to catalyze the biosynthesis of MK-4 when using VK_1_ and VK_3_ as the isopentenyl acceptor. In addition, we constructed a ribosomal DNA (rDNA)-mediated multi-copy expression vector for the fusion expression of *Sa*GGPPS and *Pp*IDI, and the recombinant GGU-GrIG afforded higher MK-4 production, so that it was selected as the high-yield strain. Finally, the yield of MK-4 was maximized at 0.24 mg/g DCW by improving the GGPP supply when VK_3_ was the isopentenyl acceptor.

**Conclusions:**

In this study, we constructed a novel synthetic pathway in *P. pastoris* for the biosynthesis of the high value-added prenylated product MK-4 through heterologous expression of *Hs*UBIAD1 and strengthened accumulation of GGPP. This approach could be further developed and accomplished for the biosynthesis of other prenylated products, which has great significance for theoretical research and industrial application.

## Background

Predominant Most research on vitamin K has been devoted to its physiological function as an essential cofactor for γ-glutamyl carboxylase (GGCX), an enzyme that catalyzes the posttranslational modification of proteins involved in blood coagulation and bone metabolism and prevention of cardiovascular calcification [1–5]. Natural vitamin K has two molecular forms: the plant form, phylloquinone (PK) or vitamin K_1_, and the bacterial form, known as the menaquinones (MKs) or vitamin K_2_ [6, 7]. The common structure of a 2-methyl-1,4-naphthoquinone nucleus is present in both PK and MKs, which are structurally different in the length and degree of saturation of the isoprene side-chain. At the 3-position of the common nucleus, PK has a monounsaturated side chain of four isoprenyl residues. In contrast, MKs are classified into 14 types, which are named MK-n according to the number of repeating unsaturated isoprenyl units. Over the past few years, numerous studies have indicated that MKs can play a role in treating mitochondrial pathologies such as Parkinson’s disease and amyotrophic lateral sclerosis, and even exhibit anticancer activity in several types of cancer cells, including liver cancer, lung cancer, bladder cancer, prostate cancer and ovarian cancer [8–11]. PK is primarily obtained from leafy green vegetables and is endogenously converted to menatetrenone (MK-4) by cleavage of the side chain in the intestine, then menadione (VK_3_) is delivered through the mesenteric lymphatic system and blood circulation to tissues such as the brain, kidney and pancreas [1, 4, 12]. Recent research has found that *Homo sapiens* UbiA prenyltransferase containing 1 (*Hs*UBIAD1), a human homologue of *Escherichia coli* prenyltransferase menA and mammalian mitochondrial prenyltransferase COQ_2_ catalyzes the conversion of PK to its prenylated derivative MK-4 [12–14]. As a member of the membrane prenyltransferase family, the structure of *Hs*UBIAD1 has been resolved in many studies; it contains eight transmembrane helices and two characteristic conserved motifs (NDxxDxxxD and DxxxD) [15, 16]. In particular, the isoprenyl substrate is located in the membrane embedded active site between the two aspartate-rich motifs. In previous studies, *Hs*UBIAD1 was usually expressed in animal cell expression systems to investigate its related properties [6, 17]. This may be because animal cell expression systems have certain advantages in expressing desired proteins with near-native functional properties. Nevertheless, we cannot ignore the shortcomings of low expression levels, high cost and time- consuming and complicated culture conditions. The components of the medium used for animal cell culture are relatively complex and expensive. In addition, animal cell culture systems are sensitive to the environment and often present the risk of infection with bacteria, fungi and viruses, resulting in relatively high requirements for the operating environment and equipment. Furthermore, it is relatively difficult to screen stable high expression strains using animal cell expression systems and often requires a longer time period due to their low recombination rate. The methylotrophic yeast, *Pichia pastoris* has become an attractive workhorse for biotechnology, especially for the production of both secreted and intracellular heterologous proteins [18–21]. It is an important heterologous protein expressing system that has several advantages over other eukaryotic and prokaryotic expression systems. Firstly, *P. pastoris* not only has the ability to grow high cell densities with a rapid growth rate, it is also capable of metabolizing methanol as its sole carbon and energy source [22, 23]. In addition, the heterologous genes of interest have been stably integrated into the genome, ideally into a targeted locus via homologous recombination that eliminates segregational instability, which is different from expression via plasmid DNA [24, 25]. Also, *P. pastoris* has the subcellular structure of eukaryotes, so that it exhibits certain advantages in post-translational modifications, including polypeptide folding, phosphorylation, glycosylation and methylation [26, 27]. In order to achieve successful recombinant protein expression, choosing the appropriate expression vector is an important prerequisite as well as selection of the host strain. Thus far, the majority of heterologous proteins have been successfully expressed by methanol inducible alcohol oxidase promoter (P_*AOX1*_) and glyceraldehyde-3-phosphate dehydrogenase constitutive promoter (P_*GAP*_). In the presence of methanol, P_*AOX1*_ is induced to generate the enzyme alcohol oxidase [28], which catalyzes the first step of formaldehyde assimilation pathways, converting methanol to formaldehyde [29, 30]. It has been reported that the expression levels of heterologous proteins are tightly regulated by P_*AOX1*_ [31, 32]. However, large amounts of methanol are needed as inducer for large-scale fermentations, resulting in risks of toxicity and safety. From a security perspective, the constitutive expression vector controlled by P_*GAP*_ is more appropriate for recombinant protein production. Furthermore, recent studies have shown that the level of expression seen with the P_*GAP*_ can be slightly higher than that obtained with the P_*AOX1*_ [29, 31, 33, 34].

**Fig. 1:**
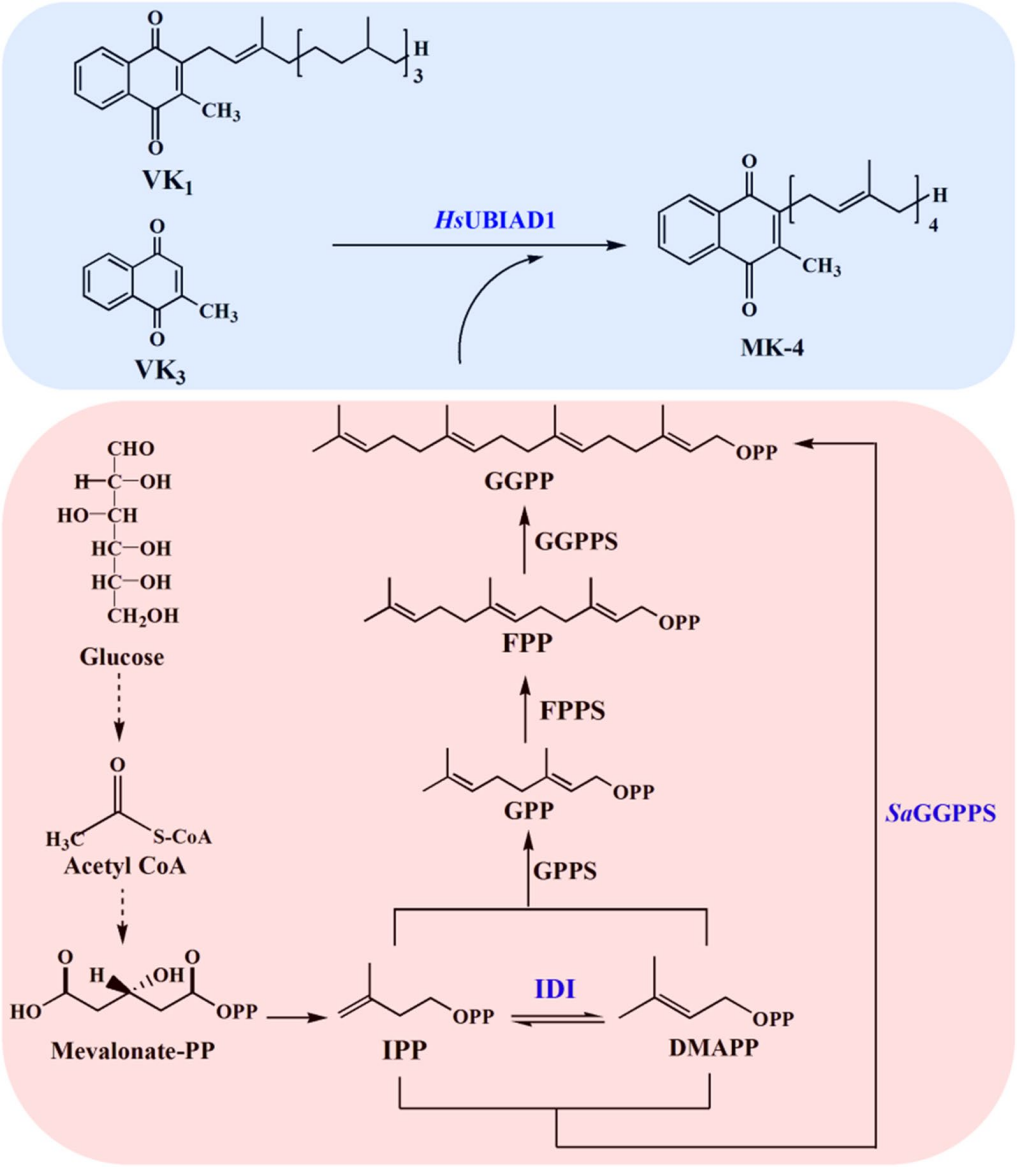
Biosynthetic pathway for MK-4 in *P. pastoris* GS115

Geranylgeranyl diphosphate (GGPP), an important precursor for the biosynthesis of MK-4, consists of isoprenoid building blocks accumulated via the mevalonate (MVA) pathway in *P. pastoris* (Fig. 1). Research has shown that the geranylgeranyl diphosphate synthase from the archaebacterium *Sulfolobus acidocaldarius* (*Sa*GGPPS) is capable of synthesizing geranylgeranyl diphosphate through sequential condensations of isopentenyl diphosphate (IPP) with dimethylallyl diphosphate (DMAPP), thereby reducing competition for the common precursor farnesyl diphosphate (FPP) [35–37].

A previous study [38] found that *P. pastoris* has much higher menadione and MKs tolerance when compared with other host strains such as *E. coli*. As an appropriate host due to its superior ability to express eukaryotic proteins, *P. pastoris* has many biotechnological applications; however, the menaquinone biosynthetic pathway has not been discovered in *P. pastoris*. Considering all of the above, in the present study we have attempted to construct a novel synthetic pathway in *P. pastoris* for the production of MK-4 by heterologous expression of *Hs*UBIAD1. Meanwhile, to increase MK-4 production, the *Sulfolobus acidocaldarius* geranylgeranyl diphosphate synthase (*Sa*GGPPS) was introduced into *P. pastoris* to improve GGPP supply, although *P. pastoris* does contain the genes that encode a GGPP synthase.

## Materials and methods

### Strains and plasmids

*Escherichia coli* DH5α cells used for plasmid propagation were preserved in our laboratory. *P. pastoris* GS115 (kindly provided by the Laboratory of Signaling Transduction and Transcription, University of Science and Technology of China, Hefei, China) was used to construct the MK-4 production strain. Expression vectors pPICZA and pGAPZA were purchased from YouBio (Hunan, China). All strains and plasmids used in this study are listed in Additional file 1: Tables S1 and S2.

### Culture conditions

*Escherichia coli* were incubated at 37 °C in a low-salt LB medium consisting of 1% tryptone, 0.5% yeast extract, and 0.5% NaCl. YPD medium (2% tryptone, 1% yeast extract, and 2% glucose) was used for cultivating *P. pastoris* for further preparation of competent cells. BMGY medium used for activating recombinant *P. pastoris* consisted of 2% tryptone, 1% yeast extract, 1.34% yeast nitrogen base (YNB) medium without amino acids, 1% glycerol, 4 × 10^−5^ % biotin, and 0.1 M potassium phosphate (pH 6.0). The constitutive recombinant *P. pastoris* with P_*GAP*_ promoter was incubated for approximately 24 h at 30 °C and 250 rpm in 250-mL flasks containing 25 mL of BMGY medium. For protein expression driven by P_*AOX1*_ in *P. pastoris*, the cells were harvested by centrifuging at 5000×*g* for 10 min at room temperature and resuspended in BMMY medium (2% tryptone, 1% yeast extract, 1.34% YNB, 4 × 10^−5^ % biotin and 0.1 M potassium phosphate (pH 6.0) and 0.3% methanol) to a final density at OD_600_ of 1.0, and methanol was added to the BMMY medium to a final concentration of 0.3% every 24 h. Solid medium was obtained by adding 2% agar to the liquid medium. The media were prepared by sterilization at 121 °C for 20 min, and glucose solution was sterilized separately at 115 °C for 15 min. In addition, the stock solution of 10 × YNB and 500× biotin were added separately after filtration sterilization.

### Bioinformatics analysis of *Hs*UBIAD1

SOPMA (https://npsa-prabi.ibcp.fr/cgi-bin/npsa_automat.pl?page=npsa_sopma.html) was applied to predict the secondary structure of *Hs*UBIAD1 from the amino acid sequence, in particular to predict the rate of secondary structural elements [39]. Since the hydrophobicity of an amino acid sequence predicts the protein folding, we analyzed the hydrophobicity of *Hs*UBIAD1 using several tools including SOSUI, ProtScale and DNAMAN. SignalP 3.0 (http://www.cbs.dtu.dk/services/SignalP-3.0/) is commonly used to predict the presence and location of signal peptide, and it was used to discriminate the signal peptide of *Hs*UBIAD1 [40]. WoLF PSORT (https://wolfpsort.hgc.jp/) was used to predict the subcellular location of *Hs*UBIAD1 based on the PSORT II program [41]. The transmembrane topology of *Hs*UBIAD1 was predicted using a range of transmembrane topology predictors, such as Phobius, OCTOPUS and TMHMM. And the online analysis software, Protter, was used to visualize the transmembrane topology of *Hs*UBIAD1. SWISS-MODEL, an automated protein homology-modelling server, was used to predict the tertiary structure of *Hs*UBIAD1 based on its amino acid sequence.

The homologous sequences of *Hs*UBIAD1 obtained from the National Center for Biotechnology Information (NCBI) database were aligned using Clustal X. Then, phylogenetic analyses were conducted using MEGA X program. The evolutionary history was inferred using the neighbor-joining method based on the Poisson model and bootstrap analysis was carried out with 1000 replicates. The phylogenetic tree was further edited by EvolView. Multiple sequence alignment of *Ap*UbiA, *At*PPT1, *Ec*UbiA, *Le*PGT-1, *Os*PPT, *Sc*CoQ_2_, *Bl*MenA, *Ec*MenA, *Kp*MenA, *Pa*UBIAD1, *Pt*UBIAD1 and *Hs*UBIAD1 were performed using Clustal X and then analyzed by ESPript 3.0.

### Construction of the expression vector

The codon of the *Hs*UBIAD1 gene, which was artificially synthesized by General Biosystems, Inc. (Anhui, China), was optimized based on codon preference to achieve high levels of protein expression in *P. pastoris*. The CAI and GC contents of the optimized sequence were analyzed by the GenScript Web server (http://www.genscript.com). The *Hs*UBIAD1 gene was amplified using primers UBIAD1-F-EcoRI and UBIAD1-R-NotI listed in Additional file 1: Table S3, which contained full-length UBIAD1. The PCR products were digested with *Eco*RI *and Not*I and inserted between the *Eco*RI and *Not*I sites of pGAPZA where *Hs*UBIAD1 is under the control of constitutive promoter P_*GAP*_ on the vector, and the resulting plasmid was named pGU. The *Hs*UBIAD1 fragment described above was cloned into multiple cloning sites of pPICZA, which thus generated pPU. In the recombinant pPU vector, *Hs*UBIAD1 protein was controlled by P_*AOX1*_. The recombinant plasmids grown on low-salt LB agar plates with 50 μg/mL Zeocin were sequenced after colony PCR to confirm the presence and orientation of the insert.

To obtain stable multi-copy integrants in *P. pastoris*, we attempted to construct a multi-copy expression vector by integrating the nucleotide sequence of a portion of the 28S ribosome DNA (rDNA) amplified from *P. pastoris* into the expression vector pGAPZA. Concretely, the rDNA fragment was amplified from the genomic DNA of *P. pastoris* GS115 using primers rDNA-F and rDNA-R listed in Additional file 1: Table S3, and cloned into the *Bam*HI site of pGAPZA to gain the desired expression vector pGAPZA-rDNA. The geranylgeranyl pyrophosphate synthase gene (*ggpps*) from *Sulfolobus acidocaldarius* used in this study was artificially synthesized by General Biosystems, Inc. (Anhui, China). The coding region of *ggpps* was amplified by PCR using primers GGPPS-F-EcoRI and GGPPS-R-NotI listed in Additional file 1: Table S3, and cloned into the expression vector pGAPZA-rDNA using the same methods, thus generating pGrG. Subsequently, the expression cassettes of *Sa*GGPPS were integrated into the rDNA locus by homologous recombination.

The IPP isomerase gene (*idi*) was PCR amplified from the genomic DNA of *P. pastoris* GS115 using primers IDI-F-EcoRI and IDI-R (Additional file 1: Table S3), while the *ggpps* fragment was PCR amplified from the plasmid pGrG by primers GGPPS-F and GGPPS-R-*Not*I listed in Additional file 1: Table S3. The *idi*-*ggpps* fusion gene was constructed by fusing the *ggpps* gene to the 3′-end of the *idi* gene as follows, and the flexible linker (GGGGS)_2_ sequence GGTGGCGGTGGCTCGGGCGGTGGTGGGTCG was inserted between the *idi* and *ggpps* genes. These two fragments were purified separately and then fused by overlap extension PCR using the primer pair of IDI-F-*Eco*RI and GGPPS-R-*Not*I (Additional file 1: Table S3), which contained EcoRI at the 5′-end and NotI at the 3′-end. The *idi*-*ggpps* fusion gene was digested with *Eco*RI *and Not*I and cloned into multicloning sites of pGAPZA-rDNA as described earlier, and the corresponding plasmid of pGrIG was then constructed.

### Transformation and screening of recombinant *Hs*UBIAD1-producing *P. pastoris*

The electrocompetent cells of *P. pastoris* GS115 were prepared according to the following protocol. The single colony of *P. pastoris* was grown in 250-mL flasks containing 25 mL of YPD medium and incubated at 30 °C and 250 rpm overnight (16–18 h). 50 mL of fresh YPD medium were inoculated in a 250-mL flask with 0.5 mL of the overnight culture until it reached an OD_600_ of 1.1–1.3. The culture was centrifuged at 1500×*g* for 5 min at 4 °C, then the cells were suspended in 20 mL of ice-cold, sterile water. The cells were centrifuged at 1500×*g* for 5 min at 0 °C, then resuspended the pellet with 20 mL of ice-cold sterile water, repeatedly. After three centrifugations, the pellet was resuspend in 5 mL of ice-cold 1 M sorbitol. After centrifugation again at 1500×*g* for 5 min at 0 °C, the cells were finally suspended in 0.2 mL of ice-cold 1 M sorbitol.

The recombinant vectors pGU and pPU were linearized by the restriction enzymes *Bsp*HI and *Sac*I separately, then electroporated into the disarmed *P. pastoris* GS115 by electroporation. Transformants were selected on MD histidine deficient plates (1.34% YNB, 4 × 10^−5^ % biotin, 2% glucose, and 2% agar) containing Zeocin (100 μg/mL), and the presence the of expression cassette was confirmed by colony PCR using pGAP-F/3′AOX1 and 5′AOX1/3′AOX1 primer pairs listed in Additional file 1: Table S3. After initial selection, a range of concentrations (200, 400, 500, 1000 μg/mL) of Zeocin was used to select multi-copy transformants.

### Expression of the recombinant *Hs*UBIAD1

The colony that was positive for GGU and GPU was cultured for 24 h at 30 °C and 250 rpm in an 18 × 180 mm test tube containing 5 mL of BMGY medium for the expression of recombinant *Hs*UBIAD1. After 24 h of cultivation, activated GPU cells were harvested and resuspended in 5 mL of BMMY medium in the same test tube for inductive fermentation at 22 °C for 48 h with the addition 2% methanol every 24 h. The intracellular proteins of recombinant *P. pastoris* were extracted using Yeast Total Protein Extraction Kit (Sangon Biotech, Shanghai, China). Then, the expression levels of recombinant *Hs*UBIAD1 were detected by dot-blot to screen the high-yield *Hs*UBIAD1-producing strains.

To obtain the optimal expression conditions in *P. pastoris* GS115, the culture temperature ranged from 20 to 30 °C, and the initial pH values ranged from 4 to 9. The recombinant *P. pastoris* was grown at 250 rpm in 250-mL flasks containing 50 mL of BMGY medium, and samples were taken every 12 h during the fermentation. Once the culture was completed, the samples were centrifuged at 13,000×*g* for 5 min at 4 °C, then the recombinant *Hs*UBIAD1 was extracted from the wet cell mass using the Yeast Total Protein Extraction Kit. Next, the expression levels were determined by western blot analysis, and ImageJ was used to process and analyze the results with β-actin as the reference. At the same time, another portion of the wet cells after centrifugation was dried in a vacuum freeze dryer for 6–8 h, and the biomass of the recombinant GGU-23 was detected by measuring the dry cell weight (DCW). All values reported are the averages of triplicate trials (± standard error).

### Western blot detection of *Hs*UBIAD1

The samples were mixed with 5× SDS-PAGE sample loading buffer and boiled for 5 min. Protein bands were separated by SDS-PAGE with a 12% separating gel and a 5% stacking gel, and transferred to nitrocellulose membranes (Beyotime, Shanghai, China). The membranes were incubated with 5% nonfat dried milk in TBS with 0.1% Tween-20 (TBST) for blocking, and then incubated with 6 × His-tag mouse monoclonal antibody (1:2500 dilution, EnoGene Biotech, Nanjing, China). After washing with TBST, the membranes were incubated with appropriate horseradish peroxidase (HRP)-conjugated goat anti-mouse IgG (1:5000 dilution, EnoGene Biotech, Nanjing, China). Finally, the immunoreactive proteins were detected with BeyoECL Star (Beyotime, Shanghai, China).

### Purification of the recombinant *Hs*UBIAD1

The intracellular proteins previously extracted were concentrated and subjected to subsequent purification using Ni–NTA affinity chromatography. The crude enzyme solution was purified with a Ni–NTA Sefinose™ Resin Kit (BBI, UK) according to the manufacturer’s specifications. Then the eluent was passed through a desalting gravity column (BBI, UK) to remove the salts. The purified proteins of *Hs*UBIAD1 were frozen and stored at − 80 °C in preparation for subsequent experiments.

### Enzymatic assay of *Hs*UBIAD1

The enzymatic reaction for *Hs*UBIAD1 was conducted in vitro and the conditions were adjusted according to the method of Hirota et al. [1]. Reactions were initially performed in 1 mL of 100 mM Tris–HCl (pH 8.0) containing 1 mM DTT, 10 μM GGPP, 10 μM VK_1_ or VK_3_ and 10 mg of *Hs*UBIAD1 protein. Reaction mixtures were incubated at 37 °C for 1 h, and then stopped by the addition of 1 mL of methanol, and the organic phase was used for HPLC analysis. The activity of *Hs*UBIAD1 was measured at different pH and temperature values in the presence of different metallic ions, and the initial activity of *Hs*UBIAD1 was measured without metal ions at pH 8.0 and 37 °C. The reported values of relative activity are the averages of triplicate trials.

Additionally, we analyzed the enzymatic reaction conditions of *Hs*UBIAD1 in vivo using the whole-cell catalytic system. After 36 h of fermentation, VK_3_ (10 mg/L, in methanol) and MgCl_2_ (10 mM) were added to the catalysis mixture, which was incubated at 31 °C for to realize the subsequent catalytic reaction. Subsequently, the catalytic products were extracted and detected.

### Transformation and screening of high-yield MK-4 producing *P. pastoris*

The recombinant plasmids pGrG and pGrIG were linearized by the restriction enzyme *SpeI* for integration into the rDNA locus, and electroporated into the electrocompetent cells of the high-yield strain GGU-23 to generate GGU-GrG and GGU-GrIG, respectively. Transformants were selected on histidine-deficient MD agar plates containing 500 μg/mL Zeocin. Then, colony PCR analysis was rapidly performed to identify the presence of the expression cassette using the pGAP-F/3′AOX1 primer pairs listed in Additional file 1: Table S3. To screen the high-yield MK-4 producing *P. pastoris*, the positive transformants GGU-GrG and GGU-GrIG were inoculated and subsequently treated as described previously.

### Extraction and analysis of MK-4

To extract a certain amount of MK-4 to be used for analysis, the recombinant *P. pastoris* was cultured according to the previous method. After fermentation and whole cell catalysis, 25 mL of fermented broth were centrifuged at 13,000×*g* for 5 min at 4 °C and dried in a vacuum freeze dryer for 6–8 h. The biomass of each recombinant was determined by weighing the freeze-dried cell mass, then methanol (5 mL) was added, followed by static extraction for 2 h. Then n-butyl alcohol (5 mL) was added to the supernatants and the extraction mixture was oscillated for 2 h at 150 rpm. The organic phase was centrifuged at 13,000×*g* for 10 min and filtered through 0.45 μm pore organic membranes. Both intracellular and extracellular MK-4 levels were analyzed by high performance liquid chromatography (HPLC). Methanol and dichloromethane were selected to be the mobile phase at a ratio of 4:1 (v/v). The detector was operated at the wavelength of 248 nm for the quantitative detection of MK-4, at which the menaquinones exhibited a strong UV absorption. The contents of MK-4 were calculated from the peak areas based on standard curves of MK-4. Then, the prenylated products were analyzed using a liquid chromatography mass spectrometry (LC–MS) system equipped with LTQ Orbitrap XL ETD analyzer (Thermo Fisher Scientific, USA).

## Results

### Bioinformatics analysis of *Hs*UBIAD1

The analysis results of SOPMA showed that *Hs*UBIAD1 consists of 49.70% alpha helices, 18.34% extended strands, 5.03% β-turns and 26.92% random coils (Additional file 1: Figure S1). The prediction results of ProtScale showed a GRVAY (Grand average of hydropathicity) value of 0.528, indicating that *Hs*UBIAD1 is a hydrophobin, which was consistent with the predictions of SOSUI (Additional file 1: Figure S2). In addition, the hydrophobicity plots generated by DNAMAN showed that significant hydrophobic domains appear in the potential transmembrane region predicted by Phobius (Additional file 1: Figure S3). The prediction results of SignalP 3.0 using neural networks (NN) and hidden Markov models (HMM) indicated that native *Hs*UBIAD1 has no signal peptide or signal anchor. In other words, *Hs*UBIAD1is a non-secretory protein. Based on the results of WoLF PSORT, *Hs*UBIAD1 was predicted to contain a KKXX-like motif (SLPK) in the C-terminus, which was regarded as an endoplasmic reticulum (ER) membrane retention signal.

*Hs*UBIAD1 was identified as a typical multi-spanning integral membrane protein according to the results of 13 transmembrane topology predictors, whereas different analysis software predicted a number of transmembrane helical segments (TMHs) ranging from 7 to 9 (Additional file 1: Table S4). Hence, the exact number of TMHs has not been determined from the current results. On the flip side, the forecasted results of most predictions indicated that the N-terminus of *Hs*UBIAD1 was located on the cytoplasmic side of the membrane (Additional file 1: Figure S4). And the tertiary structure of *Hs*UBIAD1 was predicted to contain 9 α-helices generated with the SWISS-MODEL program, using the *Ap*UbiA (*Aeropyrum pernix*, 4-hydroxybenzoate octaprenyltransferase) structure as a template (Additional file 1: Figure S5).

**Fig. 2:**
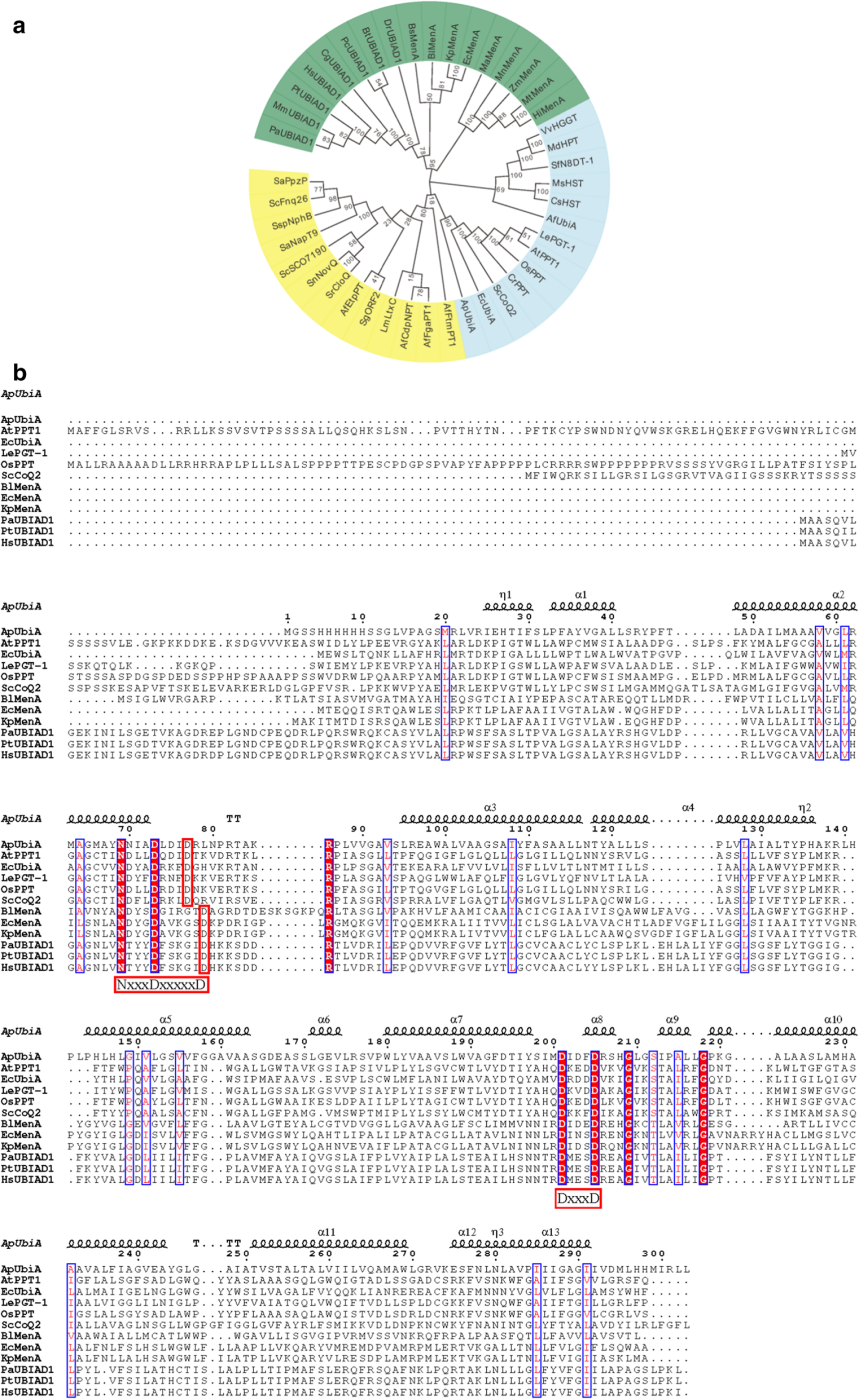
Bioinformatic analysis of the aromatic prenyltransferase (aPT) *Hs*UBIAD1. **a** Phylogenetic analysis of *Hs*UBIAD1 with the Mega X program. The phylogenetic tree was divided into two distinct clades: blue, UbiA family of membrane-bound aromatic prenyltransferases; green, menA family of membrane-bound aromatic prenyltransferases; yellow, soluble aromatic prenyltransferases. **b** Multiple sequence alignment of *Hs*UBIAD1. The two conserved aspartate-rich motifs characteristic of the membrane-bound aromatic prenyltransferases, Ap, *Aeropyrum pernix*; At, *Arabidopsis thaliana*; Ec, *Escherichia coli*; Le, *Lithospermum erythrorhizon*; Os, *Oryza sativa*; Sc, *Saccharomyces cerevisiae*; Bl, *Bifidobacterium longum*; Kp, *Klebsiella pneumoniae*; Pa, *Papio anubis*; Pt, *Piliocolobus tephrosceles*; Hs, *Homo sapiens*

The aromatic prenyltransferase (aPT) catalyzes the transfer of the isopentene group into the aromatic nucleus, and can be classified into two types, membrane-bound and soluble, according to bioinformatics analysis. The phylogenetic tree was constructed with the neighbor-joining method based on the Poisson model to show the relationship between *Hs*UBIAD1 and other aPTs. The results showed that *Hs*UBIAD1 is located in the membrane-bound clade rather than the soluble clade. Moreover, *Hs*UBIAD1 should belong to the menA family rather than the UbiA family (Fig. 2a).

Next, multiple sequence alignments were carried out among several membrane-bound aromatic prenyltransferases and the results indicated that members of the menA family contain two aspartate-rich motifs (NxxxDxxxxxD and DxxxD) (Fig. 2b). Similar to the conserved motifs of the UbiA family, the conserved domains of *Hs*UBIAD1 were presumed to be the recognition site of the isoprenyl side chain.

### Construction of the MK-4 biosynthetic pathway in *P. pastoris*

In order to improve the expression level of *Hs*UBIAD1 in *P. pastoris*, we performed a codon optimization of the original *Hs*UBIAD1. After codon optimization, 242 bases were substituted and 210 codons were modified, and the codon adaptation index (CAI) was significantly increased from 0.59 to 0.85, which is considered to be suitable for good expression of *Hs*UBIAD1 according to the GenScript Web server. The original and codon-optimized synthetic sequences of *Hs*UBIAD1 are shown in Additional file 1: Figure S6. Furthermore, for efficient initiation of translation, the Kozak sequence (GCCACC) was introduced in front of the initiation codon by primer UBIAD1-F-EcoRI.

Moreover, to compare the effects of different promoters on protein expression levels, the expression vectors pGAPZA and pPICZA were used to produce recombinant *Hs*UBIAD1, that contains the constitutive promoter P_GAP_ and the methanol inducible promoter P_AOX1_, respectively. As shown in Additional file 1: Figure S7a, the PCR product was amplified and cloned into the *Eco*RI and *Not*I sites of pGAPZA and pPICZA, to generate recombinant pGU and pPU vectors, respectively. The results of sequencing verified that the presence and correct orientation of the *Hs*UBIAD1 ORF.

### Transformation and screening of recombinant *Hs*UBIAD1 producing *P. pastoris*

The desired recombinant plasmids were obtained after selection and verification, and 5–10 μg of plasmid DNA were linearized and condensed, and resuspended in 10 μL of ultrapure water, which was electroporated into the electrocompetent cells of *P. pastoris* GS115. The expression vectors pGAPZA and pPICZA contained the *HIS4* gene and Zeocin™ resistance gene, which can be used as selectable markers for screening of recombinant *Hs*UBIAD1. The result of colony PCR and sequencing showed that the expression cassette was inserted successfully into the GS115 genome. The results of the dot-blotting assay showed that the relative expression level of *Hs*UBIAD1 in recombinant GGU was obviously superior to that of GPU (Additional file 1: Figure S8). In particular, GGU-23 (E4) gave 105.8% higher *Hs*UBIAD1 production, when compared with that of GPU-23 (E8), which was selected as the high-yield *Hs*UBIAD1-producing strain.

### Effects of culture conditions on *Hs*UBIAD1 expression level in recombinant GGU-23

**Fig. 3:**
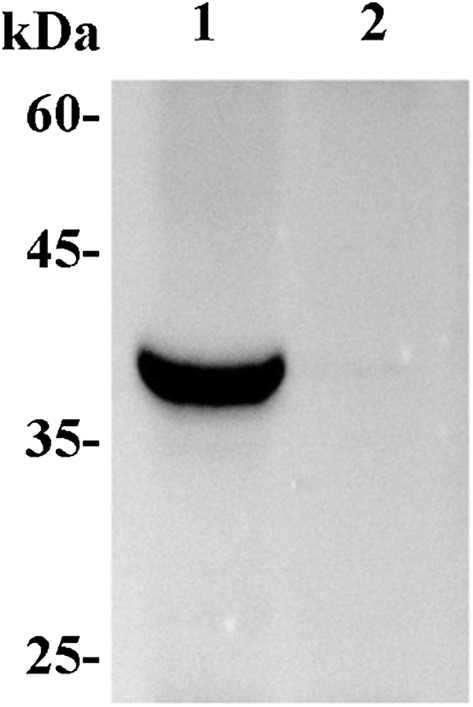
Western blot analysis of *Hs*UBIAD1 in recombinant GGU-23. Lane 1, recombinant GGU-23; Lane 2, *P. pastoris* GS115

**Fig. 4:**
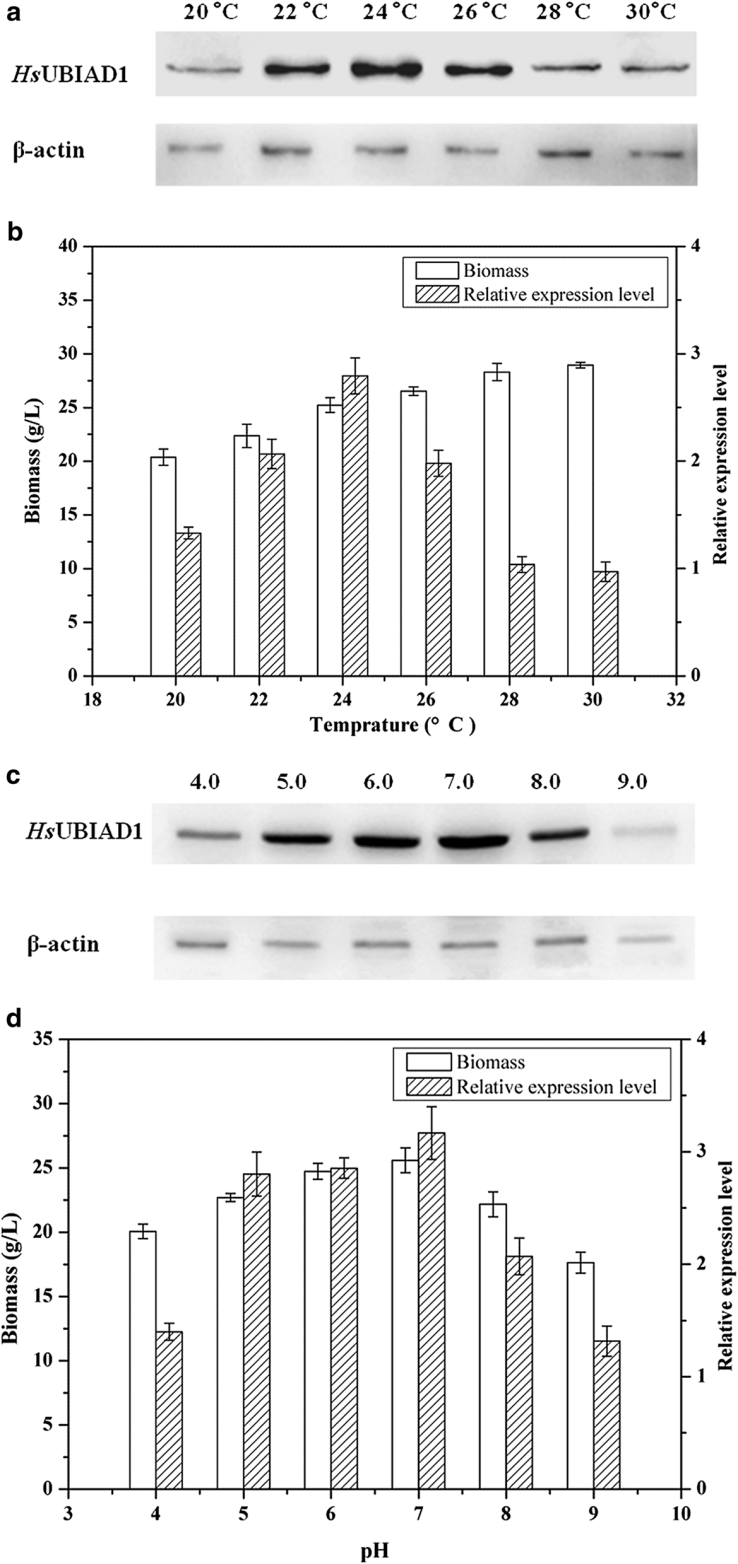

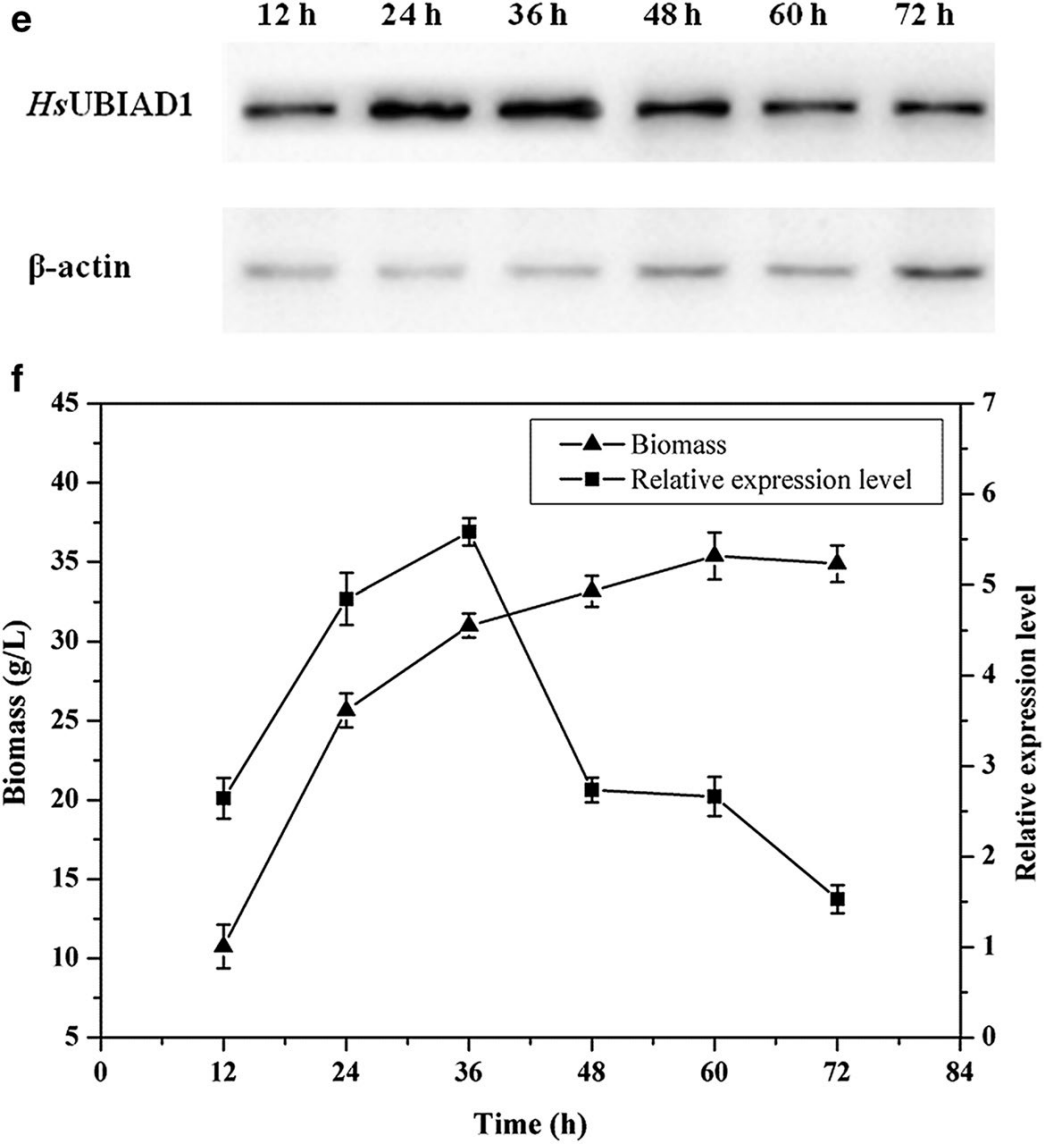
Effects of culture condition on the expression level of *Hs*UBIAD1. **a** Western blot analysis of *Hs*UBIAD1 at different temperature (at initial pH 6.0, for 24 h); **b** The expression of *Hs*UBIAD1 at different temperature (at initial pH 6.0, for 24 h); **c** Western blot analysis of *Hs*UBIAD1 at different initial pH (at 24 °C, for 24 h); **d** The expression of *Hs*UBIAD1 at different initial pH (at 24 °C, for 24 h); **e** Western blot analysis of *Hs*UBIAD1 for different time (at initial pH 7.0, at 24 °C); **f** The expression of *Hs*UBIAD1 for different time (at initial pH 7.0, at 24 °C)

The analysis of the western blot results showed a band between 35 and 45 kDa in samples from recombinant GGU-23 that was not present in the negative control (Fig. 3). And the molecular weight of native *Hs*UBIAD1 was calculated based on the aminoacid sequence as 37 kDa, which means that the exogenous *Hs*UBIAD1 was successfully expressed in the recombinant *P. pastoris* strains. To improve the expression level of *Hs*UBIAD1 in recombinant GGU-23, the initial pH and culture temperature during the fermentation processes were optimized. The production of *Hs*UBIAD1 increased by 4.37 times upon after incubation at pH 7.0 and 24 °C for 36 h, when compared with that under the initial conditions (Fig. 4).

### Enzymatic assays of *Hs*UBIAD1

**Fig. 5:**
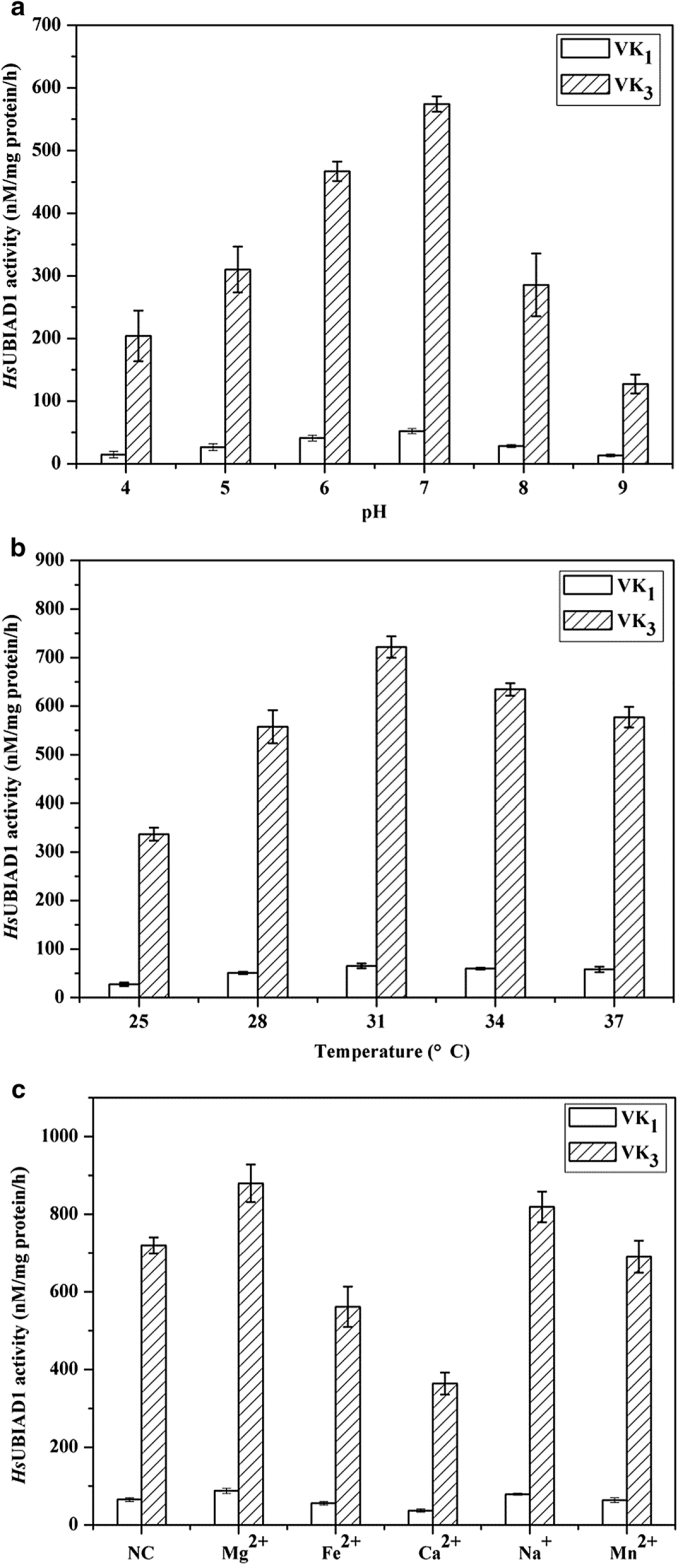
Effects of reaction condition on *Hs*UBIAD1 activity using phylloquinone (VK_1_) and menadione (VK_3_) as the donor of naphthoquinone ring. **a** Effects of pH on *Hs*UBIAD1 activity (at 37 °C, without metallic ions); **b** Effects of temperature on *Hs*UBIAD1 activity (at initial pH 7.0, without metallic ions); **c** Effects of metallic ions on *Hs*UBIAD1 activity (at pH 7.0 and 31 °C), NC, negative control, without metallic ions

In this study, the effects of pH, temperature and metal ions on the activity of *Hs*UBIAD1 in vitro were investigated. After optimization, the most appropriate pH was 7.0 and the most appropriate temperature was 31 °C for MK-4 production in the presence of Mg^2+^ (Fig. 5). Under the optimum conditions, the activity of *Hs*UBIAD1 was 229% higher than before. The results indicated that metal ions were not required for the synthesis of MK-4 catalyzed by *Hs*UBIAD1, but the addition of Mg^2+^ and Na^+^ improved the catalytic activity while Fe^2+^, Ca^2+^ and Mn^2+^ inhibited the prenylation reaction to some extent.

After 36 h of fermentation, VK_3_ was directly added as the prenyl acceptor to the fermentation broth for prolonged cultivation without changing the medium, which was incubated at 31 °C for another 18 h. In this case, it is believed that Mg^2+^ promotes the binding of substrate GGPP to *Hs*UBIAD1, although MK-4 could be biosynthesized in the absence of Mg^2+^.

### Improving GGPP supply to increase the yield of intracellular MK-4

**Fig. 6:**
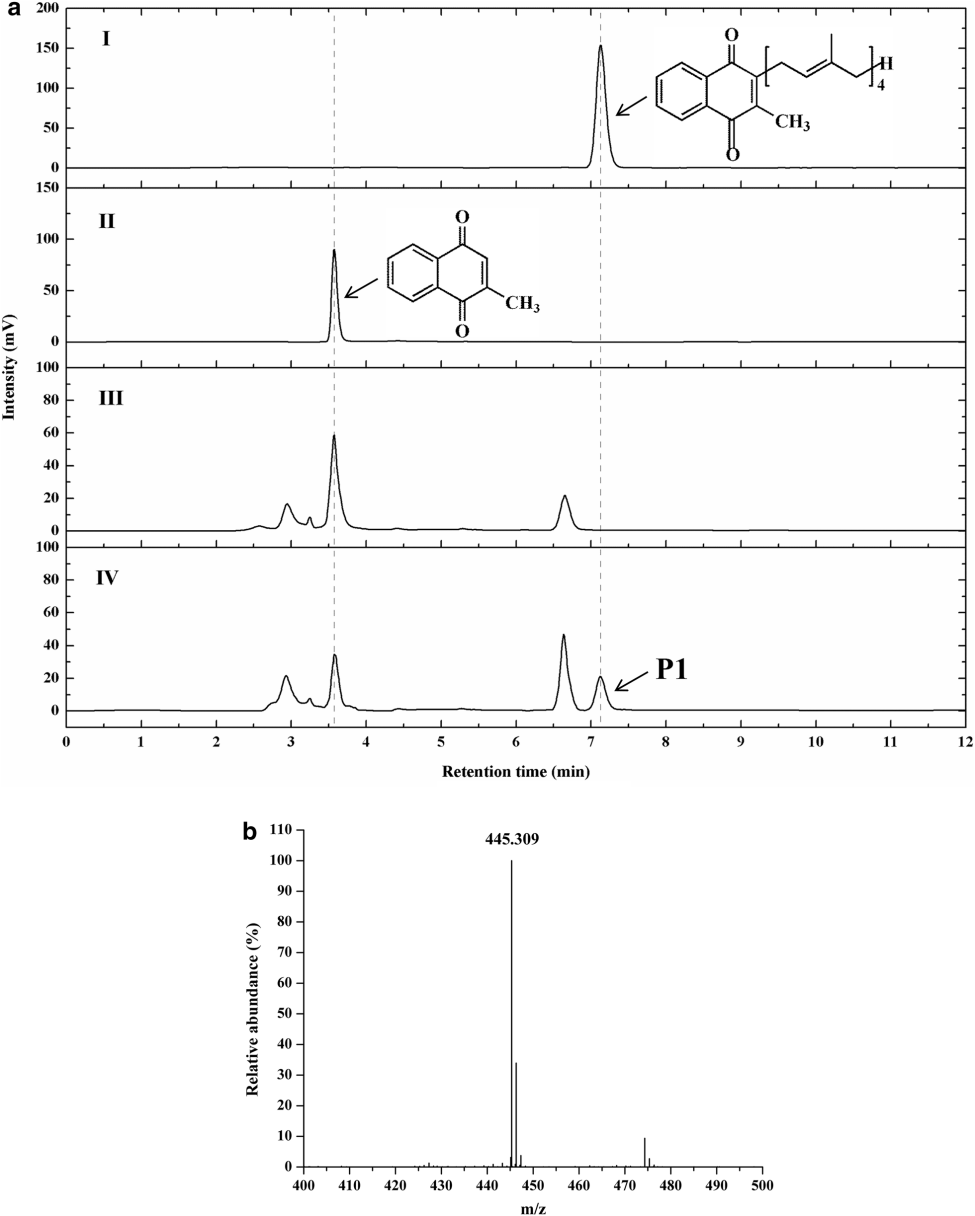
Products analysis of whole-cell catalytic prenylation. **a** HPLC analysis of prenylated products using VK_3_ as prenyl acceptor in whole-cell catalytic system. I, MK-4 standard; II, VK_3_ standard; III, Intracellular catalytic products of wild-type GS115; IV, Intracellular catalytic products of recombinant GGU-GrIG. **b** Mass spectrometric analysis of prenylated products

**Fig. 7:**
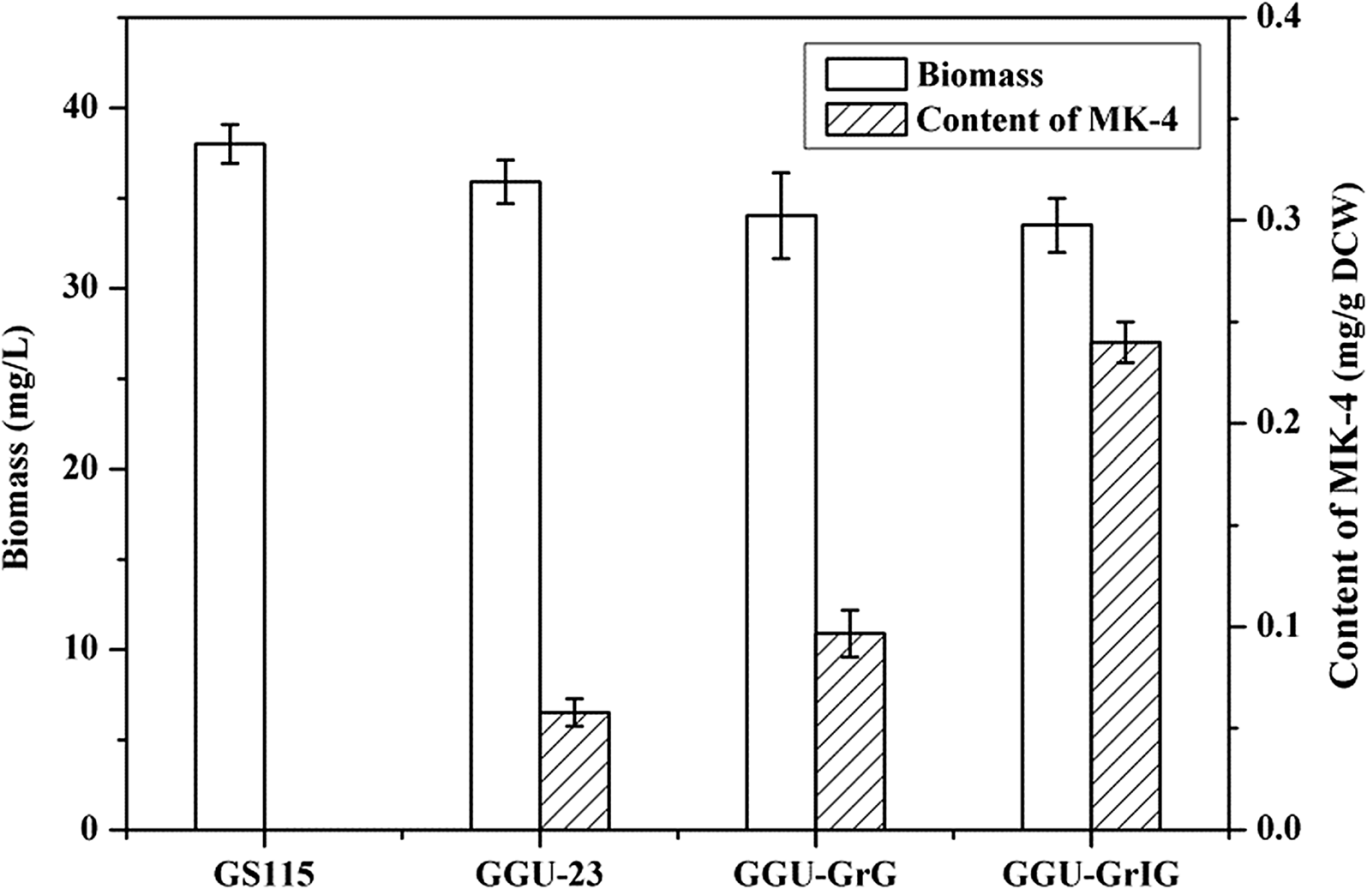
The content of MK-4 in recombinant GGU-GrIG

To further increase the production of MK-4, we attempted to improve the supply of GGPP by heterogenous expression of *Sa*GGPPS and fusion expression of *Pp*IDI and *Sa*GGPPS (Additional file 1: Figure S7b). The expression cassettes were integrated into the rDNA locus rather than the GAP locus to increase the copy number. After transformation, recombinant strains of GGU-GrG and GGU-GrIG were cultured in test tubes prior to screening the high-yield strains for MK-4 production. The results of colony PCR confirmed that either pGrG or pGrIG was present in the the genomic DNA of the recombinant GGU-23. According to the HPLC results, a novel prenylated product appeared at 7.1 min compared to wild-type GS115, which was basically consistent with MK-4 standards (Fig. 6a). The mass spectrometric analysis of the prenylated product displayed a peak with m/z = 445, which corresponds to the hydrogen adduct cation of MK-4 (Fig. 6b). It was confirmed that MK-4 was predominantly biosynthesized intracellularly but was not detected in the supernatant. Furthermore, GGU-GrIG afforded 140% higher MK-4 production, when compared with that noted in GGU-GrG (Fig. 7). The yield of intracellular MK-4 was significantly improved to 0.24 mg/g DCW when additional GGPP was supplied.

## Discussion

In recent decades, *P. pastoris* has become a mature and efficient expression system for recombinant exogenous proteins production. Above all, *P. pastoris* is easy to manipulate genetically, quickly grows to very high cell density in a relatively simple and inexpensive medium, and is able to secrete large amounts of recombinant proteins [21]. Furthermore, unlike the prokaryotic expression systems, *P. pastoris* has a eukaryotic post-translational modification system, which can facilitate expression of correctly-folded eukaryotic proteins [18, 42, 43]. And plasmids integrated into the genome by homologous recombination are not easily lost from their host, resulting in enhanced stability of the recombinant strain. However, due to the lack of native pathway for the biosynthesis of the naphthoquinone precursor, *P. pastoris* cannot synthesize menaquinone by itself. Moreover, if the de novo synthesis pathway of naphthoquinone ring is constructed, a series of enzymes need to be heterologously expressed, which involves complex regulation and time-consuming screening processes. Considering various aspects, it is more economically viable to exogenously add the naphthoquinone precursor analog VK_3_ into the reaction system than to introduce an exogenous synthetic pathway in *P. pastoris*. Therefore, we attempted to achieve the synthesis of MK-4 by heterologous expression of the key enzyme that catalyzes the polymerization of the naphthoquinone ring and isopentenyl side chain rather than a series of enzymes. In the past, many papers and much research has suggested that *Hs*UBIAD1 is an efficient MK-4 biosynthetic enzyme that may have both side-chain cleavage and prenylation activities, as it is an aPT homologous to *Ec*MenA [1, 2, 4, 6]. Previous studies have also suggested that *Hs*UBIAD1 should belong to the UbiA family. But with the deepening of research, *Hs*UBIAD1 is currently considered to belong to the menA family rather than the UbiA family, which is completely consistent with the analysis of the phylogenetic tree in this study. Besides, the discrepancy in prediction results was due to the different algorithms of the different transmembrane topology predictors. *Hs*UBIAD1 should contain 9 TMHs based on a comprehensive analysis of the transmembrane topology and hydrophobicity. This conclusion was reached for reference only, and the exact transmembrane topology of *Hs*UBIAD1 remain uncertain. Since the structure and function of proteins are closely related, predicting the physicochemical properties and structure of proteins, and analyzing their genetic relationships can lay a good theoretical foundation for the study of subsequent related protein functions and mechanisms. We confirmed that *Hs*UBIAD1 is a membrane-bound aromatic prenyltransferase by bioinformatics analysis, which affects the selection of subsequent expression and purification strategies. In this study, we successfully constructed a heterologous expression vector for the efficient expression of *Hs*UBIAD1 in *P. pastoris*. The recombinant plasmids contain the *HIS4* gene and Zeocin™ resistance gene as the selectable markers, and the *Hs*UBIAD1 fragment was integrated into the P_*GAP*_ locus of the chromosome by homologous recombination. At the same time, we also found that P_*GAP*_ was a strong constitutive promoter that can be used without methanol induction, which makes it possible to regulate the fermentation process safely and simply. Glycerol was used as the sole carbon source during the whole fermentation process to avoid adding methanol; as it was not necessary to shift cultures from one carbon source to another, strain growth was more straightforward. As an important regulatory element, the performance of the promoter directly affects the transcription level of the gene. It is gratifying that the expression level of *Hs*UBIAD1 was improved by 105.8% and the fermentation time was shortened by half under the control of P_*GAP*_ in this study.

On the one hand, the highest yield of *Hs*UBIAD1 was obtained at pH 7.0, which was suitable for biomass growth of GGU-23. The theoretical isoelectric point (pI) was calculated based on the *Hs*UBIAD1 aminoacid sequence as 8.4. Thus, it is assumed that the low expression level of *Hs*UBIAD1 may be caused by the alkaline environment, which may inhibit the correct folding and stability of the *Hs*UBIAD1 protein. On the other hand, the optimal culture temperature was 24 °C for the expression of *Hs*UBIAD1, although higher temperatures were more conducive to the growth of the cells. It is possible that low temperature culture facilitated the expression and proper folding of *Hs*UBIAD1. By optimizing the expression conditions, the production of *Hs*UBIAD1 increased by 4.37 times upon incubation at pH 7.0 and 24 °C for 36 h, when compared to the initial conditions (pH of 6.0 and induced at 28 °C for 24 h). Similar to these results, a low temperature culture has been shown to promote the expression of exogenous proteins in *P. pastoris* based on recent studies [42, 44, 45].

Herein, the results have demonstrated that *Hs*UBIAD1 expressed in *P. pastoris* has the ability to catalyze the biosynthesis of MK-4. Previous studies have shown that Mg^2+^ plays a key role in binding the side-chain of substrates to *Hs*UBIAD1 [1, 2]. However, Hirota et al. have reported that Mg^2+^ was necessary for recognizing and activating the short-chain isoprenyl substrates GPP or FPP, while the larger hydrophobic region of GGPP was directly recognized by *Hs*UBIAD1 without the participation of Mg^2+^ [6]. In the present study, we found that GGPP could be recognized by *Hs*UBIAD1 and catalyze prenyl transfer to aromatic substrates with or without the participation of Mg^2+^. In addition, *Hs*UBIAD1 displayed higher catalytic activity when VK_3_ was used as the donor of a naphthoquinone ring. It is speculated that the isopentenyl side chain could be attached directly to the naphthoquinone ring of VK_3_, while the side chain of VK_1_ needs to be removed to release VK_3_.

However, the yield of MK-4 was low, which was caused by the low content of the side-chain substrate GGPP. To improve the intracellular GGPP supply, we attempted to heterologously express *Sa*GGPPS in recombinant GGU-23. In theory, there is a dose effect between the productivity of a heterologous protein and the copy number of the target gene [28]. In other words, we could improve the expression level of the protein by increasing the gene copy number within a certain range. In *P. pastoris*, the repeated sequence of rDNA has been used as the integration site of the expression cassettes of the target gene to obtain stable multicopy strains [46]. Similar approaches were used in the present study: we obtained stable multi-copy recombinant strains by integrating the expression cassettes of *Sa*GGPPS into the 28S rDNA locus. Many studies have shown that *Sa*GGPPS has the ability to biosynthesize GGPP by directly and continuously adding isoprenoid building blocks, which avoids competition with other branched pathways for FPP [35, 47, 48]. The yield of MK-4 was indeed improved by heterologous expression of *Sa*GGPPS. On this basis, we further increased the content of GGPP by expressing *Sa*GGPPS fused with *Pp*IDI. The purpose of the fusion expression strategy was to narrow the spatial distance between *Sa*GGPPS and *Pp*IDI and to reduce the steric hindrance of the substrates, which was able to improve the transfer efficiency of the isoprenoid building blocks and to facilitate the accumulation of the key precursor GGPP. Recently, research on multi-enzyme assembly strategies has furnished an attractive and worth-while method for promoting substrate transfer between multiple enzymes. Researchers hoped to achieve cascading amplification of target metabolites by eliminating interference from unrelated metabolic pathways. Other reported microbial stains including *Escherichia coli* and *Bacillus subtilis* have been well-documented in the biosynthesis of MKs. Liu et al. changed the state of the cell membrane in *Escherichia coli*, and the extracellular MK reached 10.71 ± 0.19 mg/L after 120 h of cultivation [49]. Wang et al. improved the redox potential and the state of the cell membrane of *B. subtilis* natto, the MK-7 concentration increased to 43 mg/L in a 5-L bioreactor for 144 h [50]. Ma et al. modified the MEP pathway in *B. subtilis* to enhance the biosynthesis of MK-7, resulting in 50 mg/L MK-7 after 120 h of cultivation [51]. Compared to other reported strains that synthesize MKs by de novo synthesis, we first achieved whole-cell catalytic synthesis of MK-4 in *P. pastoris*, which took only 54 h. Ultimately, the yield of MK-4 was remarkably enhanced to 0.24 mg/g DCW using VK_3_ as the prenyl acceptor.

## Conclusion

In this study, we constructed a novel synthetic pathway in *P. pastoris* for the production of MK-4 by heterologous expression of *Hs*UBIAD1. As a versatile and efficient expression system, *P. pastoris* has been successfully applied to the stable and efficient expression of *Hs*UBIAD1. The effects of different promoters on *Hs*UBIAD1 expression levels were investigated: P_*GAP*_ was associated with a higher expression level than that obtained with P_*AOX1*_. Moreover, P_*GAP*_ also offered significant advantages in safety and simplicity. By optimizing the expression conditions, the production of *Hs*UBIAD1 increased by 4.37 times upon incubation at pH 7.0 and 24 °C for 36 h. Moreover, we potentiated the biosynthesis of the key precursor GGPP in *P. pastoris* by fusion expression of *Sa*GGPPS and *Pp*IDI, which improved the polymerization of isoprene units. The content of MK-4 was increased to 0.24 mg/g DCW by improving the GGPP supply when VK_3_ was the isopentenyl acceptor. Thus, the concept of metabolic engineering has been successfully applied to the biosynthesis of a novel high value-added prenylated product MK-4 in *P. pastoris*, which has great significance for theoretical research and industrial application. This scheme could be further developed and refined for the biosynthesis of other prenylated products.

## Supplementary information

**Additional file 1: Figure S1.** Predicted secondary structure of *Hs*UBIAD1 by SOPMA. **Figure S2.** Hydrophobicity plots of *Hs*UBIAD1 by ProtScale and DNAMAN. **Figure S3.** The topology of *Hs*UBIAD1 generated by Protter. **Figure S4.** Tertiary structure prediction of *Hs*UBIAD1. **Figure S5.** Sequence comparison between of codon-optimized *Hs*UBIAD1 and original *Hs*UBIAD1 genes. **Figure S6.** Construction of recombinant vectors. **Figure S7.** Screening for *Hs*UBIAD1 high-yield recombinant *P. pastoris*. **Table S1.** Strain used in this study and information. **Table S2.** Plasmid used in this study. **Table S3.** Primers used in this study. **Table S4.** Prediction of transmembrane topology of *Hs*UBIAD1.

## Data Availability

All data supporting the conclusions of this study are included in this article and the additional file.
