# Supplementary material for: Construction of a novel MK-4 biosynthetic pathway in Pichia pastoris through heterologous expression of HsUBIAD1
Source: Microb Cell Fact. 2019 Oct 10;18:169. doi: 10.1186/s12934-019-1215-9 (PMC6786277; doi:10.1186/s12934-019-1215-9)
Supplement: Supplementary file 1 — Additional file 1: Figure S1. Predicted secondary structure of HsUBIAD1 by SOPMA. Figure S2. Hydrophobicity plots of HsUBIAD1 by ProtScale and DNAMAN. Figure S3. The topology of HsUBIAD1 generated by Protter. Figure S4. Tertiary structure prediction of HsUBIAD1. Figure S5. Sequence comparison between of codon-optimized HsUBIAD1 and original HsUBIAD1 genes. Figure S6. Construction of recombinant vectors. Figure S7. Screening for HsUBIAD1 high-yield recombinant P. pastoris. Table S1. Strain used in this study and information. Table S2. Plasmid used in this study. Table S3. Primers used in this study. Table S4. Prediction of transmembrane topology of HsUBIAD1. [file 12934_2019_1215_MOESM1_ESM.docx]

**Additional file**

Fig. S1 Predicted secondary structure of *Hs*UBIAD1 by SOPMA. a: h, alpha helix; e, extended strand; t, beta turn; c, random coil. b: blue, alpha helix; red, extended strand; green, beta turn; purple, random coil.

Fig. S2 Hydrophobicity plots of *Hs*UBIAD1 by ProtScale and DNAMAN. a, ProtScale; b, DNAMAN.

Fig. S3 Hydrophobic analysis of the transmembrane region of *Hs*UBIAD1. Hydrophobicity plot of *Hs*UBIAD1 was generated by DNAMAN and TMHs of *Hs*UBIAD1 were predicted by Phobius.

Fig. S4 The topology of *Hs*UBIAD1 generated by Protter. The topology model illustrates that *Hs*UBIAD1 contains nine transmembrane helices with the N-terminus is orientated intracelluarly and C-terminus is orientated extracelluarly. NxxxDxxxxxD and DxxxD conserved motif was heighted by “■” and “■”, respectively.

Fig. S5 Tertiary structure prediction of *Hs*UBIAD1. The structural model was generated with the SWISS-MODEL program, using the *Ap*UbiA (*Aeropyrum pernix*, 4-hydroxybenzoate octaprenyltransferase) structure as template.

Fig. S6 Sequence comparison between of codon-optimized *Hs*UBIAD1 and original *Hs*UBIAD1 genes.

Fig. S7 Construction of recombinant vectors. **a**, Construction of recombinant vectors of *Hs*UBIAD1. **b**, Construction of recombinant vectors of *Sa*GGPPS.

Fig. S8 Screening for *Hs*UBIAD1 high-yield recombinant *P. pastoris*. **a**, Screening of high-yield *Hs*UBIAD1-producing strains by dot-blot. A1, negative control (NC), recombinant *P. pastoris* with plasmid pGAPZA; B1-F4, recombinant *Hs*UBIAD1 producing *P. pastoris* plasmids pGU; A5, NC, recombinant *P. pastoris* with plasmid pPICZA; B5-F8, recombinant *Hs*UBIAD1 producing *P. pastoris* plasmids pPU. **b**, Relative expression level of *Hs*UBIAD1 in the recombinant GGU and GPU.

Table S1 Strain used in this study and information

Table S2 Plasmid used in this study

Table S3 Primers used in this study

Table S4 Prediction of transmembrane topology of *Hs*UBIAD1. The number and the localization of TMHs of *Hs*UBIAD1 was predicted using a range of analysis software such as Protter (<http://wlab.ethz.ch/protter/start/>), Philius (<http://www.yeastrc.org/philius/pages/philius/runPhilius.jsp>), SPLIT 4.0 (<http://split.pmfst.hr/split/4/>), HMMTOP (<http://www.enzim.hu/hmmtop/html/submit.html>), OCTOPUS (<http://octopus.cbr.su.se/>), TOPCONS (<http://topcons.cbr.su.se/>), Phobius (<http://phobius.sbc.su.se/>), PSIPRED (<http://bioinf.cs.ucl.ac.uk/psipred/>), PRED-TMR (<http://athina.biol.uoa.gr/PRED-TMR/input.html>), PredictProtein (<https://www.predictprotein.org/>), SOSUI (<http://harrier.nagahama-i-bio.ac.jp/sosui/sosui_submit.html>), TMHMM (<http://www.cbs.dtu.dk/services/TMHMM/>) and DNAMAN.

Fig. S1

a.


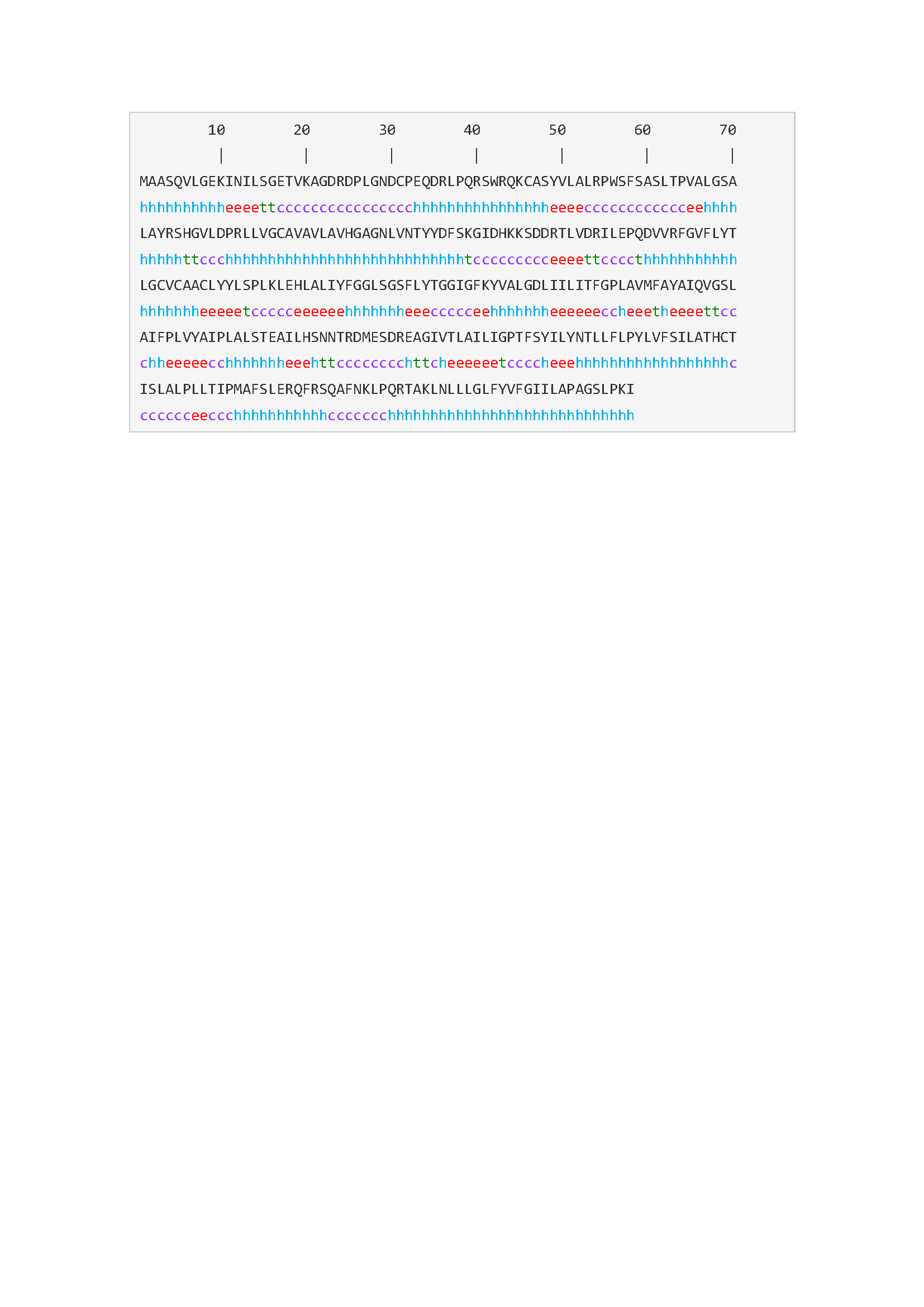


b.


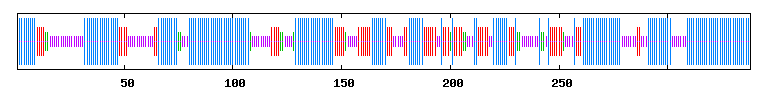


Fig. S2

a.





b.





Fig. S3





Fig. S4


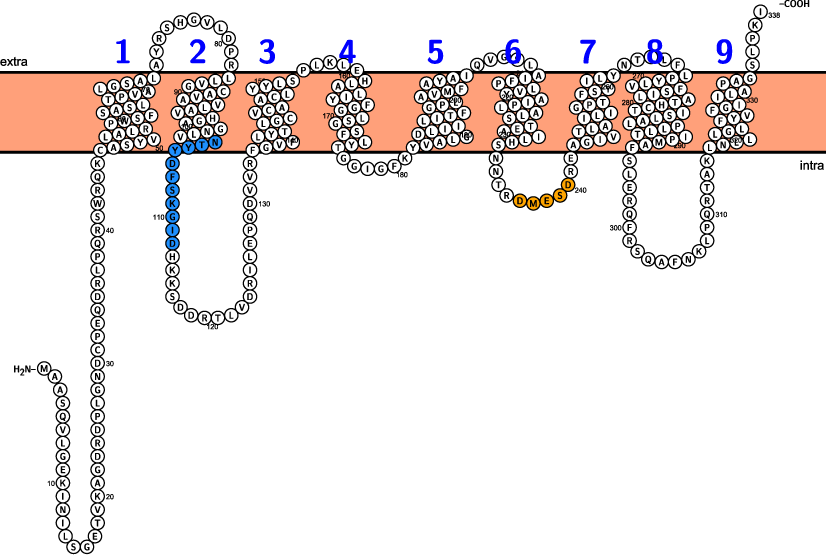


Fig. S5


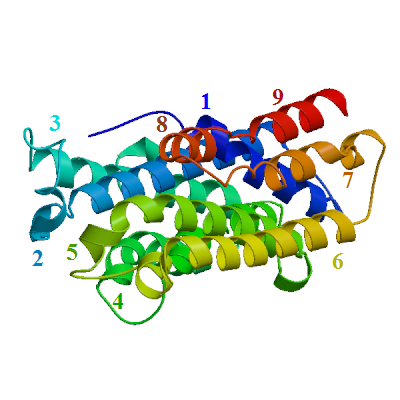


Fig. S6


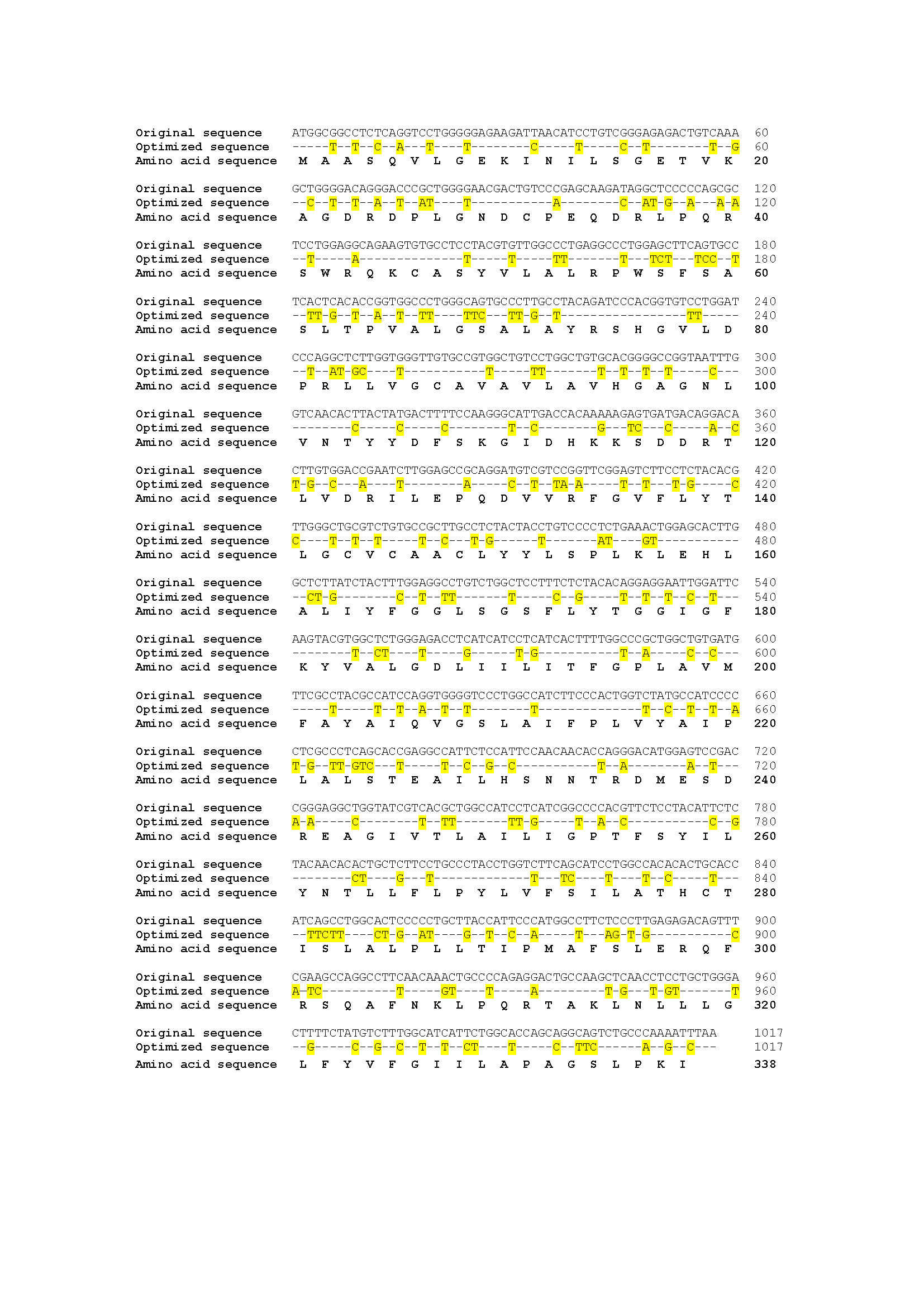


Fig. S7

a.


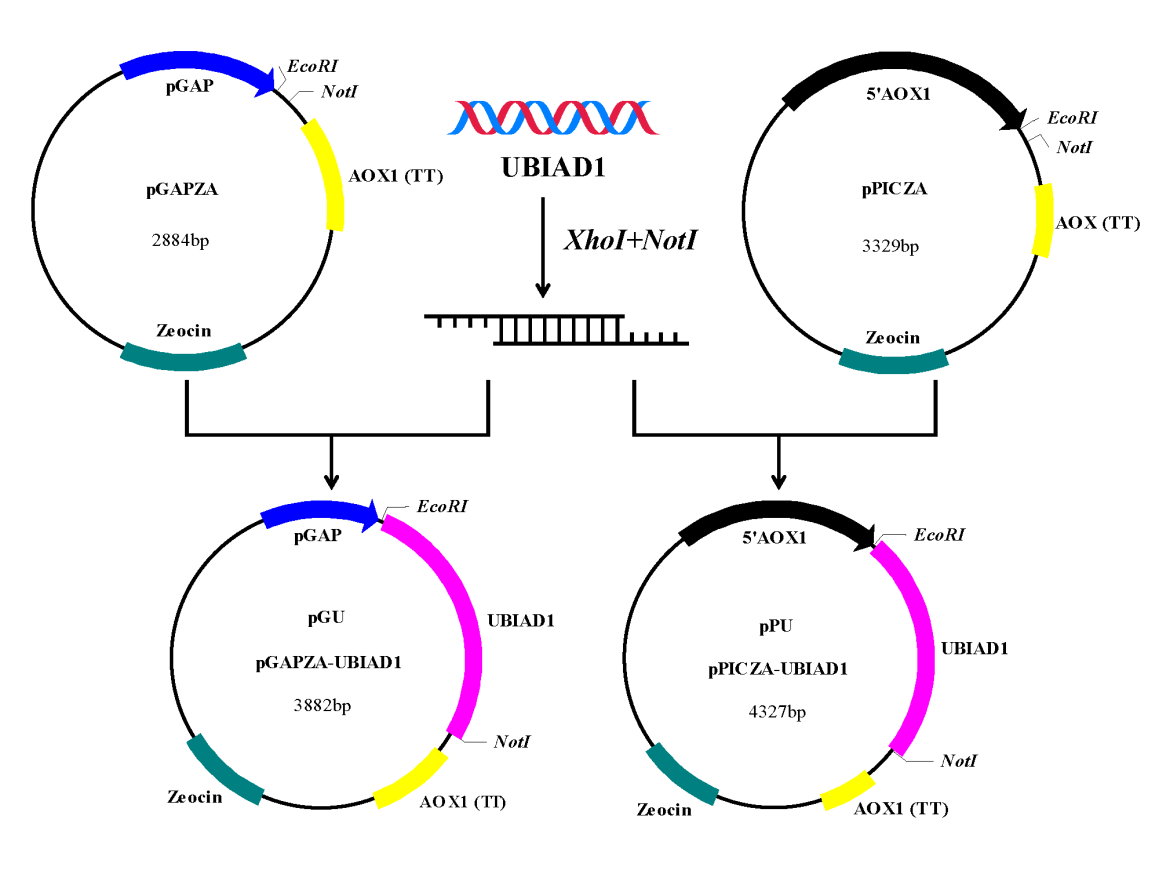


b.


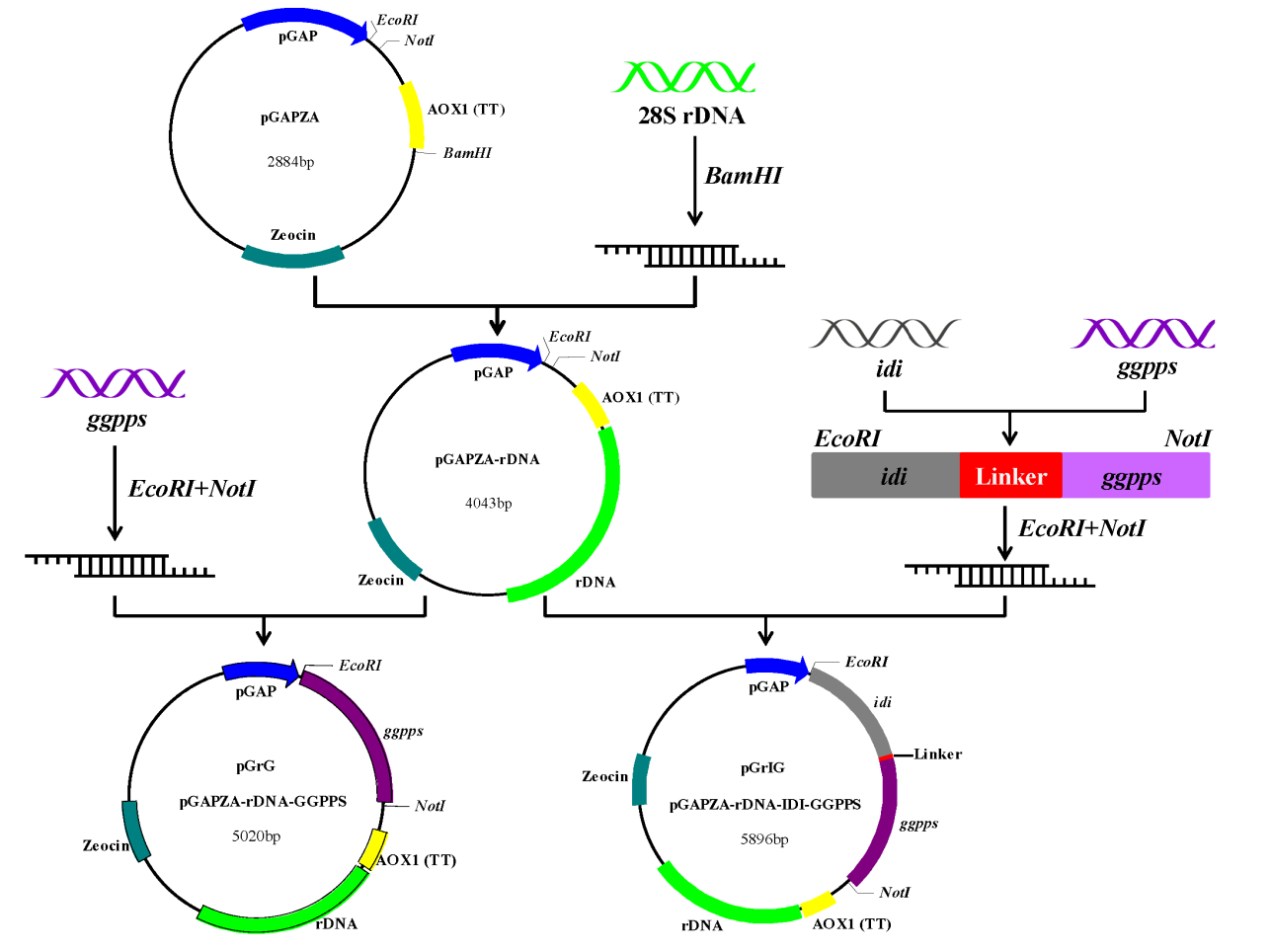


Fig. S8

a.


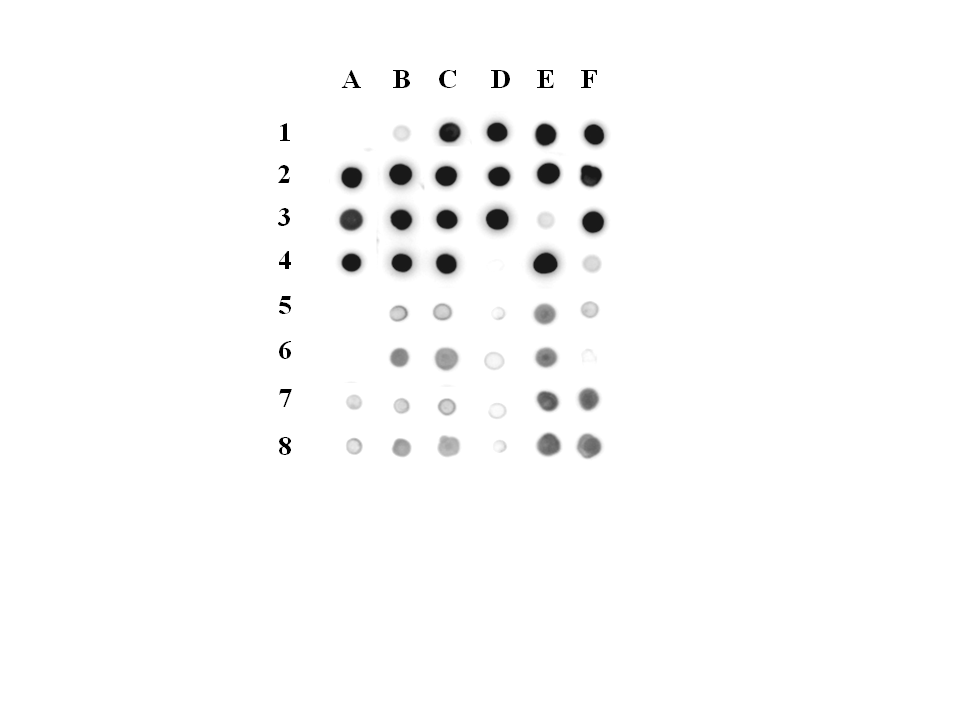


b.





Table S1

| Strain | description | reference |
| --- | --- | --- |
| *E. coli* DH5α | Host strain for plasmid propagation | Kept in our laboratory |
| *P. pastoris* GS115 | Wild type | Kept in our laboratory |
| GPU | GS115 with plasmid pPU | This study |
| GGU | GS115 with plasmid pGU | This study |
| GGU-GrG | GGU with plasmid pGrG | This study |
| GGU-GrIG | GGU with plasmid pGrIG | This study |

Table S2

| Plasmid | description | reference |
| --- | --- | --- |
| pGAPZA | Zeocin^R^, P_GAP_-based constitutive expression vector | Invitrogen |
| pPICZA | Zeocin^R^, P_AOX1_-based methanol-inducible expression vector | Invitrogen |
| pGAPZA-rDNA | Zeocin^R^, contains the nucleotide sequence of a portion of the 28S rDNA amplified from *P. pastoris*, derived from pGAPZA | This study |
| pGU | Zeocin^R^, P_GAP_-UBIAD1, derived from pGAPZA | This study |
| pPU | Zeocin^R^, P_AOX1_-UBIAD1, derived from pPICZA | This study |
| pGrG | Zeocin^R^, P_AOX1_-GGPPS, derived from pGAPZA-rDNA | This study |
| pGrIG | Zeocin^R^, Fusion of IDI and GGPPS with linker into pGAPZA-rDNA | This study |

Table S3

| Primers | Sequence (5’-3’) restriction sites are underlined |
| --- | --- |
| UBIAD1-F-EcoRI | CGGAATTCGCCACCATGGCGGCCTCTCAGGT |
| UBIAD1-R-NotI | ATAAGAATGCGGCCGCTTAATGATGATGATGATGATGAATTTTGGGCAGACTG |
| rDNA-F | CGGGATCCGTTTCGCTGGAAGGGGAACT |
| rDNA-R | CGGGATCCCAAAGACCAACCGAAAGCCGA |
| GGPPS-F-EcoRI | CGGAATTCGCCACCATGGGCAGTTACTTTGACAACTAT |
| GGPPS-R-NotI | ATAAGAATGCGGCCGCTCAATGATGATGATGATGATGTTTTCTCCTTCTTATCGT |
| IDI-F-EcoRI | CGGAATTCGCCACCATGGGTACTACGTCCGCGTATCACCAG |
| IDI-R | CACCGCCACCCAGCATACGATCAATAG |
| GGPPS-F | TATTGATCGTATGCTGGGTGGCGGTG |
| pGAP-F | GTCCCTATTTCAATCAATTGAA |
| 3’AOX1 | GGCAAATGGCATTCTGACATCC |
| 5’AOX1 | GACTGGTTCCAATTGACAAGC |

Table S4

| Method | TM1 | TM2 | TM3 | TM4 | TM5 | TM6 | TM7 | TM8 | TM9 | N-terminus location |
| --- | --- | --- | --- | --- | --- | --- | --- | --- | --- | --- |
| Protter | 46-71 | 83-105 | 134-153 | 159-175 | 182-205 | 211-231 | 243-261 | 267-294 | 315-333 | cytoplasmic side |
| Philius | 50-73 | 82-105 | 131-153 | 160-180 | 185-205 | 210-229 | 243-265 | 274-294 | 315-335 | cytoplasmic side |
| SPLIT 4.0 | 58-71 | 81-98 | 131-153 | 158-174 | 181-203 | 207-226 | 244-260 | 265-295 | 315-335 | unknown |
| HMMTOP | 46-71 | 83-101 | 132-149 | 160-179 | 188-205 | 210-229 | 250-274 | 279-296 | 317-335 | cytoplasmic side |
| OCTOPUS | 55-75 | 79-99 | 132-152 | 154-174 | 184-204 | 213-233 | 259-273 | 275-289 | 314-334 | cytoplasmic side |
| TOPCONS | 54-74 | 81-101 | 131-151 | 155-175 | 185-205 | 211-231 | 249-269 | 271-291 | 313-333 | cytoplasmic side |
| Phobius | 46-71 | 83-105 | 134-153 | 159-175 | 182-205 | 211-231 | 243-261 | 267-294 | 315-335 | cytoplasmic side |
| PSIPRED | 54-71 | 83-101 | 131-154 | 158-174 | 182-207 | 211-230 | 258-276 | 280-295 | 314-331 | cytoplasmic side |
| PRED-TMR | 55-72 | 83-101 | 134-152 | 160-180 | 188-205 | 207-225 | 248-267 | 270-290 | 315-332 | unknown |
| PredictProtein | 57-74 | 82-100 | 130-153 | 158-175 | 184-205 | 214-231 | 255-276 | 281-298 | 312-331 | unknown |
| SOSUI | 78-100 | 131-153 | 156-178 | 182-204 | 208-230 | 247-269 | 278-300 | 313-335 |  | unknown |
| TMHMM | 83-105 | 131-153 | 160-182 | 202-224 | 245-267 | 272-294 | 315-334 |  |  | cytoplasmic side |
| DNAMAN | 73-101 | 127-147 | 154-174 | 181-201 | 245-265 | 272-292 | 313-335 |  |  | unknown |
